# Ovarian Hormone-Dependent Effects of Dietary Lipids on APP/PS1 Mouse Brain

**DOI:** 10.3389/fnagi.2019.00346

**Published:** 2019-12-19

**Authors:** Jose Luis Herrera, Lara Ordoñez-Gutierrez, Gemma Fabrias, Josefina Casas, Araceli Morales, Guadalberto Hernandez, Nieves G. Acosta, Covadonga Rodriguez, Luis Prieto-Valiente, Luis M. Garcia-Segura, Francisco G. Wandosell, Rafael Alonso

**Affiliations:** ^1^Departamento de Ciencias Médicas Básicas, Instituto de Tecnologías Biomédicas–Centro de Investigaciones Biomédicas de Canarias, Universidad de La Laguna, San Cristóbal de La Laguna, Spain; ^2^Centro de Biología Molecular “Severo Ochoa” (CSIC-UAM), Universidad Autónoma de Madrid, Madrid, Spain; ^3^Centro de Investigación Biomédica en Red de Enfermedades Neurodegenerativas, Madrid, Spain; ^4^Instituto de Química Avanzada de Cataluña (IQAC-CSIC), Barcelona, Spain; ^5^Departamento de Biología Animal, Edafología y Geología, Instituto de Tecnologías Biomédicas–Centro de Investigaciones Biomédicas de Canarias, Universidad de La Laguna, San Cristóbal de La Laguna, Spain; ^6^Unidad de Estadística Médica, Universidad Católica de Murcia, Murcia, Spain; ^7^Instituto Cajal, CSIC, Madrid, Spain; ^8^Centro de Investigación Biomédica en Red de Fragilidad y Envejecimiento Saludable, Madrid, Spain

**Keywords:** cerebral cortex lipidome, long-chain polyunsaturated fatty acids, docosahexaenoic acid, sphingolipids, ovarian hormones, synaptic proteins, amyloid, estradiol

## Abstract

The formation of senile plaques through amyloid-β peptide (Aβ) aggregation is a hallmark of Alzheimer’s disease (AD). Irrespective of its actual role in the synaptic alterations and cognitive impairment associated with AD, different therapeutic approaches have been proposed to reduce plaque formation. In rodents, daily intake of omega-3 (n-3) long-chain polyunsaturated fatty acids (LC-PUFAs) is required for neural development, and there is experimental and epidemiological evidence that their inclusion in the diet has positive effects on several neurodegenerative diseases. Similarly, estradiol appears to reduce senile plaque formation in primary mouse cell cultures, human cortical neurons and mouse AD models, and it prevents Aβ toxicity in neural cell lines. We previously showed that differences in dietary n-6/n-3 LC-PUFAs ratios modify the lipid composition in the cerebral cortex of female mice and the levels of amyloid precursor protein (APP) in the brain. These effects depended in part on the presence of circulating estradiol. Here we explored whether this potentially synergistic action between diet and ovarian hormones may influence the progression of amyloidosis in an AD mouse model. Our results show that a diet with high n-3 LC-PUFA content, especially DHA (22:6n-3), reduces the hippocampal accumulation of Aβ_1__–__4__0_, but not amyloid Aβ_1__–__42_ in female APPswe/PS1 E9A mice, an effect that was counteracted by the loss of the ovaries and that depended on circulating estradiol. In addition, this interaction between dietary lipids and ovarian function also affects the composition of the brain lipidome as well as the expression of certain neuronal signaling and synaptic proteins. These findings provide new insights into how ovarian hormones and dietary composition affect the brain lipidome and amyloid burden. Furthermore, they strongly suggest that when designing dietary or pharmacological strategies to combat human neurodegenerative diseases, hormonal and metabolic status should be specifically taken into consideration as it may affect the therapeutic response.

## Introduction

Alzheimer’s Disease (AD) is characterized by the presence of senile plaques and neurofibrillary tangles in the brain, which ultimately lead to progressive neuronal dysfunction and dramatically impaired cognitive performance (Duyckaerts, 2003). It is accepted that the formation of senile plaques is a consequence of amyloid-β (Aβ) polymerization, a peptide derived from the Amyloid Precursor Protein (APP: Selkoe, 2002). According to the amyloidogenic theory, Aβ-induced toxicity leads to a cascade of events that ultimately produce oxidative stress, mitochondrial dysfunction, and synaptic impairment (Hardy and Selkoe, 2002; Gibson and Huang, 2005). A small proportion of AD cases have a genetic origin, classified as Familiar Alzheimer Disease (FAD), caused by mutations in the APP (Mullan et al., 1992), Presenilin 1 (Sherrington et al., 1996) or Presenilin 2 (Levy-Lahad et al., 1995) genes. By contrast, more than 95% of AD cases are sporadic, which implies some allelic variation in the normal population, and that aging and environmental or dietary factors influence this pathology. Indeed, although the relationship with synaptic dysfunction remains unclear, AD has been associated with metabolic disorders, such as diabetes, hypercholesterolemia or hyperlipidemia (Ricciarelli et al., 2012; Park et al., 2013; Dar et al., 2014; Baglietto-Vargas et al., 2016; Kandimalla et al., 2017; Pugazhenthi et al., 2017; Salas and De Strooper, 2019). In this respect, brain insulin resistance and IRS-1 dysfunction has been detected in AD patients (Talbot et al., 2012), and diabetes drugs represent promising therapies for AD (Ferreira et al., 2018; Boccardi et al., 2019).

Some long-chain polyunsaturated fatty acids (LC-PUFAs) like docosahexanoic acid (DHA, 22:6n-3) and pro-resolving lipid mediators (SPMs) like neuroprotectin D1 (NPD-1) or resolving D5 (RvD5), modulate signaling processes mediated by a variety of receptors, such as lipoxin A_4_ receptor, G protein-coupled receptor 32, chemerin receptor 23, and leukotriene B4 receptor, which regulate cell survival, neuroinflammation, neurotransmission, and cognitive performance (Bannenberg and Serhan, 2010; Orr et al., 2013; Serhan and Chiang, 2013; Serhan, 2014). In humans, diets enriched in LC-PUFAs have been associated with a lower incidence of dementia and neurological disorders (Mazza et al., 2007), while in rodents, n-3 LC-PUFA poor diets, such as those with limited DHA, have been associated with cognitive impairment (Ikemoto et al., 2001; Catalan et al., 2002). By contrast, high levels of DHA reduce the total Aβ and senile plaque formation, protecting AD mouse models from neuronal pathologies (Calon et al., 2004; Lim et al., 2005; Green et al., 2007; Perez et al., 2010) and improving spatial memory and cognition (Hooijmans et al., 2009). Aβ peptides are generated from the transmembrane APP through the sequential activity of two membrane associated proteases, BACE and γ-secretase. Thus, such cleavage is likely to be strongly influenced by the lipidic milieu and membrane trafficking (Hicks et al., 2012; Das et al., 2013, 2016). Several epidemiological studies suggest that dietary composition influences brain function, and specifically, that certain LC-PUFAs exert protective effects in neurodegenerative disorders (Kalmijn et al., 1997; Barberger-Gateau et al., 2002; Engelhart et al., 2002; Morris et al., 2005; Scarmeas et al., 2006; Cederholm and Palmblad, 2010; Shinto et al., 2014). Furthermore, the results of a randomized, double-blind, placebo-controlled clinical trial suggested that administration of n-3 LC-PUFAs may prevent AD, although this does not represent a treatment for the disease (Freund-Levi et al., 2006).

One issue that has not been completely addressed in all these earlier studies is that of “gender.” Human population studies have shown a clear increase in the risk of suffering AD and other dementias in women after menopause (Manly et al., 2000), suggesting a potential neuroprotective role for ovarian hormones. In rodents, the ovarian hormone 17β-estradiol (estradiol) regulates brain plasticity and cognition (reviewed in Barha and Galea, 2010), and support for its role as a neuroprotectant has been obtained in cellular models of Aβ-induced neurotoxicity (Xu et al., 1998; Marin et al., 2003; Guerra et al., 2004). In addition, both inhibition and knockout of aromatase, the enzyme that converts testosterone to estradiol, make AD pathology in animal models worse (McCullough et al., 2003; Overk et al., 2012). Furthermore, aromatase gene polymorphisms may affect the risk of AD in humans, particularly in women (Iivonen et al., 2004; Huang and Poduslo, 2006; Janicki et al., 2013; Medway et al., 2014). Taken together, all these data strongly suggest that the presence of circulating gonadal hormones may have a neuroprotective effect against several neurological diseases, although this phenomenon has not been adequately considered in the context of dietary interventions.

We previously found that different concentrations of n-6 and n-3 LC-PUFAs in the diet are associated with differences in the total fatty acids (FAs), sphingolipids, and gangliosides in the female mouse cerebral cortex. Interestingly, these changes in the brain lipidome composition are partially dependent on the presence or absence of circulating ovarian hormones (Herrera et al., 2018). Furthermore, we also found that diet and ovarian function interact and affect the expression of several neuronal proteins as well as the APP levels in the brain. Here, we set out to determine whether the intake of several of the most common n-3 LC-PUFAs, such as 20:5n-3 (EPA) and DHA, modifies the brain lipidome, the expression of neuronal proteins, and the levels of Aβ. In addition, we analyzed whether the effects of the dietary interventions were influenced by the presence or absence of circulating estradiol. To this end, we used the APPswe/PS1ΔE9 (APP/PS1) double transgenic mouse model of cerebral amyloidosis, in which increased Aβ levels develop by 6–8 months of age (Trinchese et al., 2004) as well as early synaptic defects (Viana da Silva et al., 2016). These animals were fed two experimental diets: (a) a diet with a particularly high content of 18:2n-6 PUFAs and a high n-6/n-3 PUFA ratio, also lacking DHA and other common n-3 and n-6 LC-PUFAs (DI); or (b) a diet with a lower n-6/n-3 PUFA ratio, particularly enriched in n-3 LC-PUFAs, DHA, and EPA (DII). The effects of these diets on the brain lipidome and Aβ levels were compared with that of standard laboratory food (SF) that lacks LC-PUFAs but contains the LC-PUFA precursors linoleic acid (18:2n-6) and linolenic acid (18:3n-3). At 90 days of age, all the mice were divided into three groups: (i) Sham-operated controls; (ii) Ovariectomized-placebo treated (OVX); and (iii) Ovariectomized-estradiol treated (OVX-E). After 3 months under these dietary and hormonal regimes, all mice were sacrificed and their brains were dissected out and analyzed to determine the amyloid burden, lipidome composition, and key synaptic and survival signaling proteins. Our findings support three major conclusions. First, diets containing different n-6 and n-3 PUFA ratios and LC-PUFA levels modify the cerebral cortex lipidome in female APP/PS1 mice distinctly, although the effects are not identical to those previously observed in wild type (WT) animals. Second, a diet with high levels of DHA and other LC-PUFAs reduces the hippocampal levels of amyloid Aβ1–40 in this AD model. Third, the effects of diet on the brain lipidome, amyloid burden and the expression of neuronal proteins are partially dependent on normal ovarian function. Together, these results reveal an interaction of diet and ovarian hormones that affects Aβ accumulation, lipidome composition, and neuronal protein expression in the brain of a mouse model of AD. This new information may be useful in the search for novel therapeutic interventions to either prevent or treat neurodegeneration in aging women.

## Materials and Methods

### Animals and Husbandry

Specific pathogen-free mice of two inbred strains were used in this study. Male APPswe/PS1ΔE9 (APP/PS1) and female C57BL/6J mice (*Mus musculus*) were purchased from Jackson Laboratories and Charles River Laboratories, respectively. Animals were housed under conditions of constant temperature (22 ± 2°C) and humidity (50 ± 5%), and on a 12-h light-dark cycle with free access to SF (A03/R03, SAFE-Panlab) and tap water. Crosses from both strains were genotyped by polymerase chain reaction (PCR) using DNA from tail biopsies (Jankowsky et al., 2001). Female APP/PS1 mice were selected for the experiments and housed (10 per cage) in cages fitted with microbarrier filter tops. 1 month after birth the SF was gradually replaced with the experimental diets (see below) at a rate of 25% per week. Thus, all mice were fed 100% experimental diets from the age of 2 months until the day of sacrifice (Table 1). The protocols used in this study were all approved by the Animal Care and Use Committee at University of La Laguna.

**TABLE 1:** Timeline of animal husbandry, feeding and experimental manipulations.

**Months**	**Animal manipulations**
0	After birth mice were housed under constant temperature (22 ± 2°C) and humidity (50 ± 5%) and a 12:12 h light-dark cycle, with free access to standard laboratory food
1	From day 30 of age, standard laboratory food was gradually replaced with the experimental diets at a rate of 25% per week. This dietary regime was maintained until the time of sacrifice at the age of 6 months.
3	At 90 days of age, mice were bilaterally ovariectomized or sham-operated and implanted with pellets containing 90-day timed released 17β-estradiol (0.05 mg) or vehicle
6	All mice were sacrificed at the age of 6 months

### Genotyping

The animal’s genotype was confirmed using three primers: one antisense primer matching a sequence within PrP (5′GTG GAT ACC CCC TCC CCC AGC CTA GAC C3′); one sense primer specific for the transgene (PS1, 5′CAG GTG GTG GAG CAA GAT G3′; APP, 5′CCG AGA TCT CTG AAG TGA AGA TGG ATG3′); and a second sense primer specific for the genomic PrP (5′: CCT CTT TGT GAC TAT GTG GAC TGA TGT CGG3′).

### Ovariectomy and Estradiol Treatment

Mice were anesthetized with inhaled isoflurane (2 ± 0.5%) after receiving an analgesic injection (buprenorphine hydrochloride, Buprex) and then ovariectomized bilaterally at 90 ± 1 days of age through a 1 cm dorsal incision that was then closed with surgical clips (Table 1). The day after ovariectomy, mice received a 3 mm pellet containing 0.05 mg estradiol on a 90-day time-release or a placebo (Innovative Research of America, Sarasota, FL, United States). The hormonal dose was chosen from preliminary experiments in which the levels of estradiol in 90-day-old treated animals were 7.2 ± 4.5 pg/ml (mean ± SEM), as determined by radioimmunoassay (RIA) (Architect System, ref #B7K720, Abbot, Germany). These levels are within the range of those observed in cycling animals of the same age at proestrus (20.9 ± 6.0 pg/ml) and estrus (1.8 ± 4.5 pg/ml). The pellets were implanted subcutaneously in the subscapular region using a trocar, following the manufacturer’s instructions, and no inflammation around the implantation area was observed after 3 days. Mice were then housed according to the different types of diet and hormone treatment until the end-point, 90 days later.

### Diets

Two specific experimental diets were used in these experiments (Table 2). The high n-6/n-3 ratio diet (DI) contained sunflower oil and consequently, it was particularly abundant in linoleic acid (18:2n-6) but poor in α-linolenic acid (18:n-3), with undetectable levels of EPA (20:5n-3) and DHA (22:6n-3). The low n-6/n-3 ratio diet (DII) had the same basic composition but it was specifically supplemented with extra EPA and DHA (7 g/kg), added as a lipid source in the form of fish oil to give a particularly high DHA content. These diets were designed at the “Instituto de Nutrición y Tecnología de los Alimentos” of the University of Granada (Spain), and produced by Mucedola (Mucedola srl, Milano, Italy). Both these diets and the SF were subsequently analyzed to determine the final percentage and absolute quantity (g/kg) of each FA. For simplicity, high and low n-6/n-3 PUFA ratio diets are referred to in the text and graphics as DI and DII, respectively. Mice were fed these diets *ad libitum* for 90 days until the day of sacrifice.

**TABLE 2:** Main fatty acid (FA) composition of experimental diets (DI and DII) and standard food (SF).

**Fatty acids**	**SF**	**DI**	**DII**
C 14: 0	0.05 ± 0.00	0.21 ± 0.01	1.50 ± 0.00
C 16: 0	4.13 ± 0.14	4.68 ± 0.31	6.33 ± 0.04
C 16:1 n-7	0.09 ± 0.01	0.17 ± 0.01	1.55 ± 0.01
C 18:0	0.75 ± 0.04	1.55 ± 0.08	1.77 ± 0.01
C 18:1 n-9	13.15 ± 0.35	12.04 ± 0.81	6.15 ± 0.05
C 18:1 n-7	0.34 ± 0.06	0.59 ± 0.07	0.98 ± 0.02
C 18:2 n-6	10.63 ± 0.78	28.84 ± 0.42	16.23 ± 0.00
C 18:3 n-3	1.01 ± 0.13	0.15 ± 0.03	0.44 ± 0.00
C 18:4 n-3	0.00 ± 0.00	0.00 ± 0.00	0.66 ± 0.01
C 20:0	0.11 ± 0.01	0.18 ± 0.00	0.24 ± 0.00
C 20:1 n-9	0.18 ± 0.00	0.12 ± 0.00	1.09 ± 0.07
**C 20:4 n-6 (ARA)**	0.00 ± 0.00	0.00 ± 0.00	0.20 ± 0.00
C 20:4 n-3	0.00 ± 0.00	0.00 ± 0.00	0.24 ± 0.01
**C 20:5 n-3 (EPA)**	0.00 ± 0.00	0.00 ± 0.00	2.29 ± 0.02
C 22:0	0.18 ± 0.01	0.10 ± 0.01	0.13 ± 0.02
C 22:1 n-11	0.05 ± 0.02	0.10 ± 0.06	0.74 ± 0.04
**C 22: 5 n-6 (DPA)**	0.00 ± 0.00	0.00 ± 0.00	0.11 ± 0.01
C 22: 5 n-3	0.05 ± 0.00	0.06 ± 0.03	0.43 ± 0.01
**C 22: 6 n-3 (DHA)**	0.00 ± 0.00	0.00 ± 0.00	3.47 ± 0.01
**TOTALS**			
Saturates	5.42 ± 0.16	6.83 ± 0.37	10.41 ± 0.06
Monoenes	14.00 ± 0.39	13.41 ± 1.03	11.24 ± 0.09
PUFAs	11.69 ± 0.91	29.05 ± 0.47	24.07 ± 0.08
n-9	13.47 ± 0.33	12.35 ± 0.86	7.85 ± 0.10
n-6	10.63 ± 0.78	28.84 ± 0.42	16.55 ± 0.09
n-3	1.06 ± 0.13	0.22 ± 0.06	7.52 ± 0.01
n-3 LC-PUFAs	0.05 ± 0.00	0.06 ± 0.03	6.43 ± 0.02
n-6 LC-PUFAs	0.00 ± 0.00	0.00 ± 0.00	0.32 ± 0.01
**RATIOS**			
n-3/n-6	0.10 ± 0.01	0.01 ± 0.00	0.45 ± 0.00
n-6/n-3	10.04 ± 0.52	138.37 ± 33.71	2.20 ± 0.02
Total FA content	31.53 ± 1.67	49.84 ± 2.10	46.43 ± 0.45
% Lipids (fresh wt)	5.48 ± 0.11	6.45 ± 0.55	6.31 ± 0.00
% Moisture	9.79 ± 0.09	8.65 ± 0.27	7.85 ± 0.00
% Lipids (dry wt)	6.07 ± 0.12	7.06 ± 0.59	6.85 ± 0.00

*Data are mean ± SD of g/kg of fresh weight. Totals include some minor components not shown. The most common LC-PUFAs mentioned in the text are bold marked.*

### Tissue Processing and Sample Preparation

Ninety days after ovariectomy or sham operation, the mice were sacrificed using CO_2_ and their brain was collected. For ELISAs, hippocampal tissue was homogenized in eight volumes of ice-cold guanidine buffer containing 5 mol/L guanidine HCl, 50 mmol/L Tris HCl [pH 8]. Homogenates were then mixed for 3 h at room temperature and stored at −20°C. Cerebral cortex tissue for Western blotting was homogenized in three volumes of ice-cold lysis buffer containing 20 mM HEPES, 100 mM NaCl, 100 mM NaF, 1 mM Na_3_VO_4_, 5 mM EDTA, 1% Triton X-100, 1 μM Okadaic acid (Calbiochem) and a protease inhibitor cocktail (Roche Diagnostic). The homogenates were processed as described previously (Herrera et al., 2018) and all samples were probed in 3–4 Western blots. For lipid analysis, the tissues were homogenized at a concentration of 5 mg/ml in phosphate-buffered saline (PBS) with 0.01% 3,5-Di-tert-4-butylhydroxytoluene (BHT) as an antioxidant, and they were processed as described below.

### Lipid Analysis

Lipids were extracted from dietary samples and cerebral tissue using a modification of Folch’s method (Folch et al., 1957). Dietary FA profiles (g FA/kg diet fresh weight) were obtained by acid-catalyzed transmethylation of the lipid fractions followed by GC-MS (Gas Chromatography-Mass Spectrometry: Fabelo et al., 2014). Cerebral cortex lipid fractions were subjected to further analysis of the FAs and complex lipids as described previously (Cingolani et al., 2014). Sphingolipids were analyzed by HPLC-MS using 0.2 nmol of C17-sphinganine, N-dodecanoylsphingosine, N-dodecanoylglucosylsphingosine, and N-dodecanoyl sphingosylphosphorylcholine as internal standards. Sphingolipids were annotated as <lipid subclass><total fatty acyl chain length>: <total number of unsaturated bonds>. If the sphingoid base residue was dihydrosphingosine, the lipid class contained a ð prefix. In most cases, the final tissue data were given as pmol/mg of protein, except in the case of the total FA that was represented as pmol equivalents per mg of protein with respect to C12 ceramide. For statistical analysis, the brain levels of specific LC-PUFAs were shown as the percentage of the total FA.

### Amyloid Aβ Measurement

Commercial ELISA kits were used to measure the Aβ1–40 and Aβ1–42 (A*β*_1_–_40_ human ELISA kit, KHB 3482 and Aβ_1_–_42_ human ELISA kit KHB 3442, respectively; Invitrogen, Thermo Fisher Scientific, Waltham, MA, United States). Briefly, brain homogenates were diluted 1:50 in PBS-Tween-BSA buffer (0.03% Tween-20, 5% BSA in PBS) before centrifuging at 4°C for 20 min at 16,000 × *g*. Each assay was run in duplicate and the plate absorbance was measured at 450 nm using an Opsys MR microplate reader (Dynex Technologies).

### Western Blot Analysis

Cerebral cortex protein lysates (20 μg) were resolved by SDS-PAGE, using 8 or 12% polyacrylamide gels, depending on the molecular weight of the studied proteins, and transferred onto nitrocellulose (Whatman) or PVDF (Millipore) membranes, that were subsequently blocked by incubation in 10% non-fat milk for 1 h at room temperature and incubated overnight at 4°C with the appropriate primary antibodies (Table 3) in the conditions described previously (Herrera et al., 2018). Antibody binding was detected with Supersignal (Thermo Fisher Scientific) using β-actin as the internal loading control. Densitometric analysis was performed using a GS-800 Calibrated Densitometer (Bio-Rad) and the raw data obtained are presented in Supplementary Table 1.

**TABLE 3:** Antibodies used in Western blot analysis of brain signaling and synaptic proteins.

**Antibody**	**Host**	**Dilution**	**References**
PSD95	rabbit	1:1,000	#3450 Cell Signaling, United States
PI3K-p85	mouse	1:1,000	#06-195 Upstate (Millipore), United States
Phospho-synapsin (Ser9)	rabbit	1:1,000	#2311 Cell Signaling, United States
Synapsin	rabbit	1:1,000	#2312 Cell Signaling, United States
Akt (PKB)	rabbit	1:1,000	#9272 Cell Signaling
GSK-3α/β	mouse	1:1,000	44-610 Invitrogen, United States
Phospho-GSK-3 (Ser21/9)	rabbit	1:1,000	#9331 Cell Signaling, United States
GFAP	rabbit	1:1,000	G5601 Promega, United States
Synaptophysin (SY38)	mouse	1:20,000	10701 Progen, United Kingdom
β-Actin	mouse	1:10,000	A5441 Sigma-Aldrich, United States
Goat anti-mouse IgG-HRP	goat	1:5,000	Sc-2005 Santa Cruz Biotech, Germany
Goat anti-rabbit IgG-HRP	goat	1:5,000	Sc-2004 Santa Cruz Biotech, Germany

### Statistical Analysis

To address whether different dietary n-6/n-3 ratios of LC-PUFA modify the brain lipidome in intact female APP/PS1 mice, one-way ANOVA was followed by linear contrasts, the diet being the experimental factor with three levels (SF, DI, and DII). Although the actual values of the total FAs, Ceramides, dh-Ceramides, Sphingomyelins, and dh-Sphingomyelins are represented in the corresponding figure, due to the extremely large standard errors, a logarithmic transformation was used to perform the statistical analysis. For the general ANOVA, only a *p* value < 0.05 was considered to reflect a significant effect of dietary composition. To avoid undesirable effects of multi-testing due to significance inflation, no more than two contrasts were tested for each variable. Thus, the effect of each experimental diet (DI or DII) on each specific lipid class was compared to that of the SF.

To analyze the effect of diet and the potential influence of hormonal status on the hippocampal levels of Aβ1–40 and Aβ1–42 and on protein expression in the cerebral cortex, a 2 × 3 factorial analysis was designed. Again, the two levels of the dietary factors were DI and DII, and the three levels of the gonadal factor were normal ovarian function (SHAM) and the presence (OVX-E) or absence (OVX) of circulating estradiol in ovariectomized mice. After a general ANOVA analysis, we performed no more than three contrasts to answer two specific questions: (i) for each diet, DI or DII, did the absence of the ovaries induce a significant effect; (ii) whether the presence of circulating estradiol could counteract any effect of ovariectomy; and (iii), whether the effect of ovariectomy and estradiol could be considered different when one diet was maintained as opposed to the effect of the other. In addition, to estimate the effects and interactions between different factors, we run a saturated regression model that included the dichotomous variable diet and the polytomous variable hormone condition, with three levels (Armitage et al., 2004). The latter enters the model as two dummy variables, the first with a value of “1” in ovariectomized animals receiving placebo containing pellets (OVX), and the second with a value of “1” in ovariectomized mice treated with estradiol (OVX-E). Intact sham-operated controls had a value of “0” for each dummy variable. This model provides us with confidence intervals and tests the effects of the three diets conditioned to the hormonal status and the interaction between the two factors. This means four comparisons were planned “*a priori*” in the experimental design.

## Results

### The Composition of the Diets

The composition of the experimental diets (DI and DII) was compared with that of the SF and the three diets did not differ significantly in their total lipid dry weight, ranging from 6.1% in SF to 7.1% in DI and 6.9% in DII. The total FA content (g/kg fresh weight) was also similar in DI (49.84) and DII (46.43), and it was not significantly higher than in the SF (31.53). However, the three diets did differ in terms of the content of several relevant lipid species, such as the LC-PUFAs C20:4n-6 (ARA), C20:5n-3 (EPA), C22:5n-6 (DPA), or C22:6n-3 (DHA). Neither SF nor DI presented measurable traces of these dietary constituents while they were all present in DII especially EPA and in particular, DHA. Consequently, there were dramatic differences in the dietary ratios of n-3/n-6 (SF, 0.10; DI, 0.01; DII: 0.45) and n-6/n-3 PUFAs (SF, 10.04; DI, 138.37; DII: 2.20). In summary, the main dietary differences were: a) DI had a n-6/n-3 ratio 13–14 times higher than that of SF and 59 times higher than that of DII; b) DII had a n-6/n-3 ratio 4–5 times lower than that of SF; and c) DI had no traces of either DHA or EPA, while DII contained relevant amounts of both n-3 LC-PUFAs (Table 2).

### Effect of the Different Diets on the Brain Lipidome of Female APP/PS1 Mouse According to Their Gonadal Status

**FIGURE 1:**
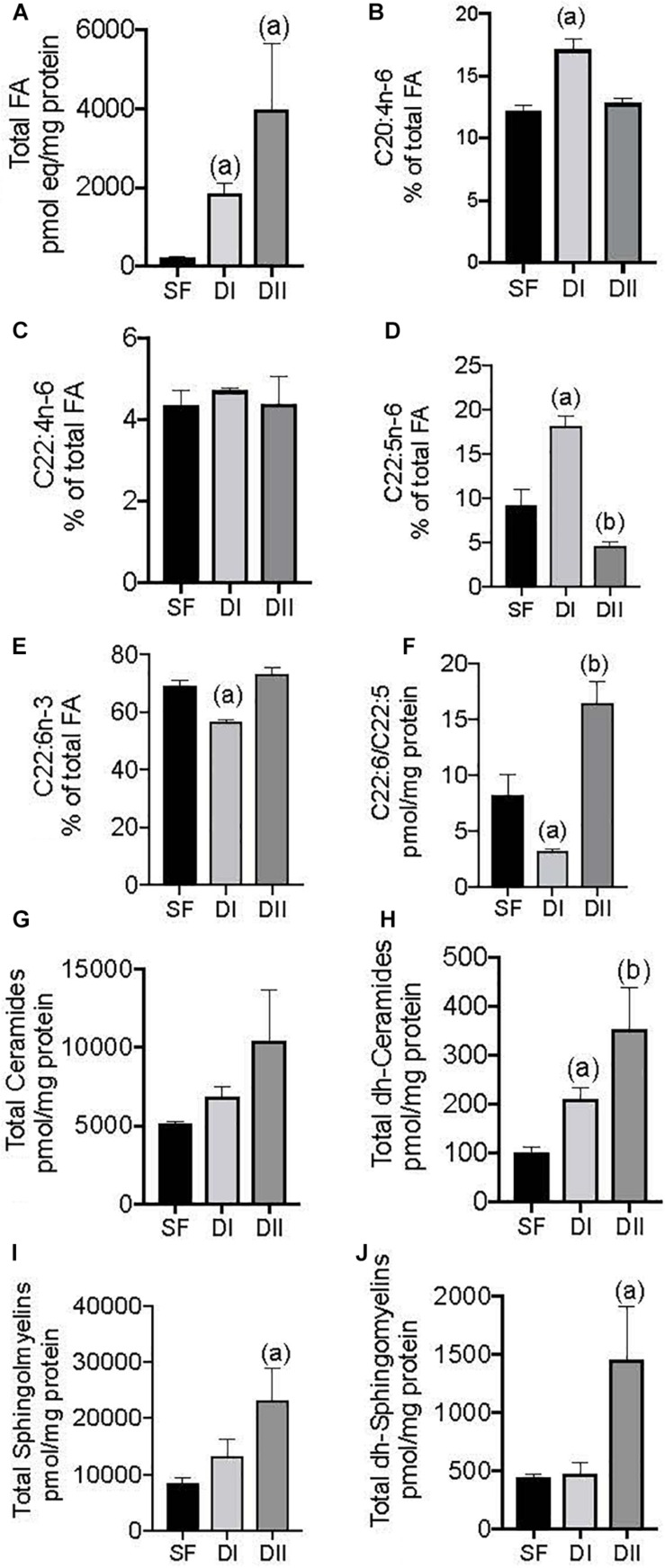
Effect of different dietary composition on cerebral cortex lipidome in intact female APP/PS1 mice. SF: Standard laboratory food (SF); DI: High n-6/n-3 ratio; DII: Low n-6/n-3 ratio. Data are represented in the units indicated in the vertical axis as mean ± SEM of four mice per group. In this and other figures, the symbols indicate significant differences obtained from the statistical analysis as described in methods section. **(A)** General ANOVA: *p* = 0.0004; (a): *p* = 0.0005 vs. SF; **(B)** General ANOVA: *p* = 0.001; (a): *p* = 0.0009 vs. SF or DII; **(C)** General ANOVA: *p* = 0.84; **(D)** General ANOVA: *p* = 0.0001; (a): *p* = 0.0005, and (b) *p* = 0.02 vs. SF and DII; **(E)** General ANOVA: *p* = 0.0004; (a) *p* = 0.001 vs. SF; **(F)** General ANOVA: *p* = 0.0005; (a) *p* = 0.05, and (b) *p* = 0.005 vs. SF; **(G)** General ANOVA: *p* = 0.18; **(H)** General ANOVA: *p* = 0.005; (a) *p* = 0.02, and (b) *p* = 0.002 vs. SF; **(I)** General ANOVA: *p* = 0.03; (a) 0.04 vs. SF; **(J)** General ANOVA: *p* = 0.01; (a): *p* = 0.007 vs. SF.

In terms of the brain lipidome, the effects of the experimental diets were compared with the SF (Figure 1). In intact sham-operated female APP/PS1 mice, the diets with different ratios of n-6/n-3 PUFAs and n-3 LC-PUFA induced distinct changes in the cerebral cortex lipidome depending on the lipid class analyzed. Animals fed either DI or DII had significantly more total FAs than animals fed SF, 10- to 20-fold more on average (Figure 1A, *p* = 0.0005). DI also induced a significant increase in the relative levels of ARA (Figure 1B, *p* = 0.0009) and DPA (Figure 1D, *p* = 0.0005) compared to SF, as well as a significant reduction in DHA (Figure 1E, *p* = 0.001) and in the DHA/DPA ratio (Figure 1F, *p* = 0.05). By contrast, DII caused a significant decrease in DPA (Figure 1D, *p* = 0.02) and a significant increase in the DHA/DPA ratio (Figure 1F, *p* = 0.005). Conversely, both the DI and DII diets significantly increased the total dh-Ceramides (Figure 1H, *p* = 0.02 and *p* = 0.002, respectively), while DII produced a significant elevation in the levels of total Sphingomyelins (Figure 1I, *p* = 0.04) and dh-Sphingomyelins (Figure 1J, *p* = 0.007).

**FIGURE 2:**
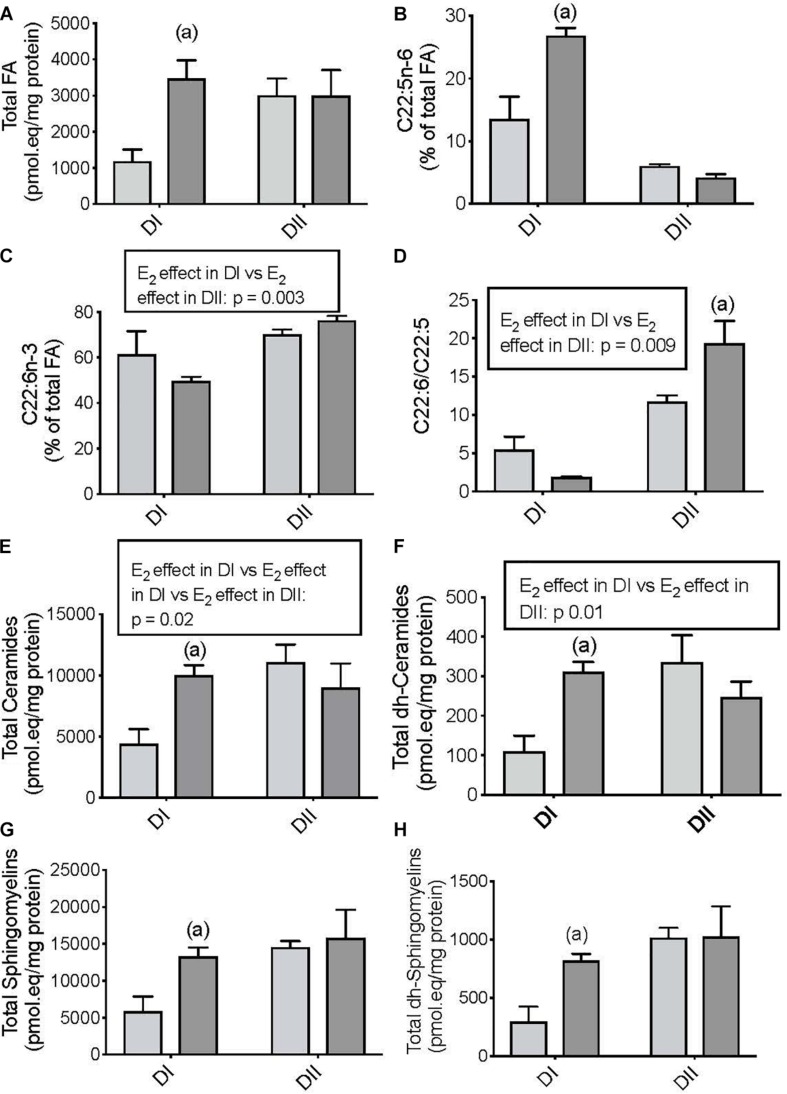
Differential effects of chronic estradiol administration on dietary-dependent cerebral cortex lipidome in ovariectomized female APP/PS1 mice. Clear bars: ovariectomized-placebo treated (OVX); Dark bars: ovariectomized-estradiol treated (OVX-E). Data are represented in the units indicated in the vertical axis as mean ± SEM of four mice per group. **(A)** General ANOVA: *p* = 0.05; (a) *p* = 0.02 vs. OVX; **(B)** General ANOVA: *p* = 0.00001; (a) *p* = 0.0001 vs. OVX; **(C)** General ANOVA: *p* = 0.0001; **(D)** General ANOVA: *p* = 0.0001; (a): *p* = 0.009 vs. OVX; **(E)** General ANOVA: *p* = 0.05; (a): *p* = 0.02 vs. OVX; **(F)** General ANOVA: *p* = 0.03; (a): 0.01 vs. OVX; **(G)** General ANOVA: *p* = 0.07; (a): *p* = 0.05 vs. OVX; **(H)** General ANOVA: *p* = 0.04; (a) *p* = 0.05 vs. OVX.

In addition to the differential effects of the diets, we also studied the influence of gonadal status on dietary-dependent brain lipidome remodeling. As such, we compared the differences between the effects of DI and DII on the cerebral cortex lipidome in OVX and OVX-E mice. In ovariectomized APP/PS1 mice fed DI, estradiol administration increased the total FAs (Figure 2A, *p* = 0.02), the relative levels of DPA (Figure 2B, *p* = 0.0001), and the total levels of Ceramides (Figure 2E, *p* = 0.02), dh-Ceramides (Figure 2F, *p* = 0.01), Sphingomyelins (Figure 2G, *p* = 0.05), and dh-Sphingomyelins (Figure 2H, *p* = 0.05). In ovariectomized mice fed DII, the only relevant effect of estradiol administration was the elevation of the DHA/DPA ratio relative to the animals receiving the placebo (Figure 2D, *p* = 0.009). We also compared the differential effect of estradiol in the animals fed DI or DII using linear saturated models. The influence of estradiol on brain lipidome remodeling depended on each specific diet, and it was found to be significant in the case of DHA (Figure 2C, *p* = 0.003), the DHA/DPA ratio (Figure 2D, *p* = 0.009), the total Ceramides (Figure 2E, *p* = 0.02), and the total dh-Ceramides (Figure 2F, *p* = 0.01).

In summary, these findings indicate that a different dietary n-6/n-3 PUFA ratios and n-3 LC-PUFA content caused specific modifications in the brain lipidome of female APP/PS1 mice, which is partially dependent on the circulating levels of estradiol.

**FIGURE 3:**
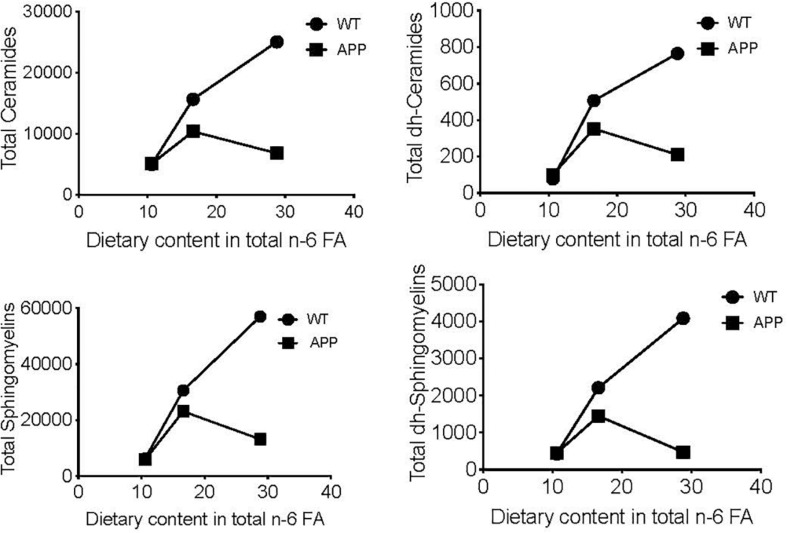
Linear regression between dietary content in n-6 FA and cerebral cortex levels of several complex lipids. Vertical axis represents the means of each compound in pmol/mg protein of four animals per group. Horizontal axis represents the progression of n-6 FA dietary content in gr/kg fresh weight (SF: 10.63; DII: 16.55; DI: 28.84). Significant dose-response effects are shown in intact WT mice for Ceramides (*p* = 0.00001), dh-Ceramides (*p* = 0.0004), Sphingomyelins (*p* = 0.0001), and dh-Sphingomyelins (*p* = 0.0001) but not in intact APP/PS1 mice. In addition, all slopes were significantly different between WT and APP/PS1 animals for Ceramides (*p* = 0.001), dh-Ceramides (*p* = 0.01), Sphingomyelins (*p* = 0.001), and dh-Sphingomyelins (*p* = 0.0009).

To visualize the differential effects of these dietary regimes in female mice with different genotypes, we compared the relationship between the total n-6 FA content of the three diets used (SF, 10.63; DII, 16.55; DI, 28.84) and the mean cerebral cortex levels of some complex lipids in WT and APP/PS1 mice. Significant dose-response effects were seen in WT animals for Ceramides (*p* = 0.00001), dh-Ceramides (*p* = 0.000), Sphingomyelins (*p* = 0.00001), and dh-Sphingomyelins (*p* = 0.00001, Figure 3). By contrast, no relationship between these two variables was detected in APP/PS1 mice for any of the lipids analyzed. In addition, all the regression slopes were significantly different in the WT and APP/PS1 mice for Ceramides (*p* = 0.001), dh-Ceramides (*p* = 0.01), Sphingomyelins (*p* = 0.001), and dh-Sphingomyelins (*p* = 0.0009).

### How of the Interaction of the Different n-6/n-3 PUFA Ratios or n-3 LC-PUFA Content and the Gonadal Status Affects Hippocampal Accumulation of Amyloid Peptide Aβ_1_–_40_ in Female APP/PS1 Mouse

**FIGURE 4:**
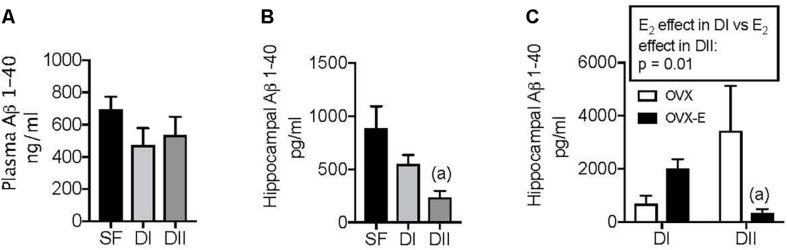
Effect of different dietary composition on plasma and hippocampal levels of A*β*_1__–__40_ in female APP/PS1 mice and its dependence on gonadal status. Data are represented in the units indicated in the vertical axis as mean ± SEM of five to six mice per group. **(A)** General ANOVA: *p* = 0.34; **(B)** General ANOVA: *p* = 0.048; (a) *p* = 0.017 vs. SF; **(C)** General ANOVA: *p* = 0.058; (a) *p* = 0.01 vs. OVX.

Changes in the dietary DHA intake reduce the amyloid burden in the brain of AD mouse models (Lim et al., 2005; Green et al., 2007), and we previously showed that different concentrations of n-6 and n-3 LC-PUFAs in the diet modify the brain levels of APP in WT mice (Herrera et al., 2018). Therefore, we analyzed the impact of both dietary composition and gonadal status on brain amyloid accumulation in APP/PS1 animals. The effects of the experimental diets on amyloid levels were compared with that of SF and initially, no significant differences in the levels the Aβ1–40 amyloid peptide were observed in the plasma with the three diets (Figure 4A, *p* = 0.34). However, there was less Aβ1–40 in the hippocampus of intact sham-operated females when animals were fed DII rather than SF (Figure 4B, *p* = 0.017). While DI appeared to produce a similar effect, the difference between the DI and SF mice was not significant. Likewise, chronic estradiol administration did not significantly affect the hippocampal amyloid accumulation in ovariectomized animals fed DI relative to those that received the placebo. By contrast, there was a significant reduction of hippocampal Aβ1–40 in OVX-E mice fed DII relative to the OVX animals (Figure 4C, *p* = 0.01). Furthermore, the influence of this gonadal hormone on amyloid levels depended on the diet, since the effect of estradiol in animals fed DI differed significantly from that observed in the mice that received DII (Figure 4C: *p* = 0.01). In contrast to Aβ1–40, no significant differences were detected in the levels of Aβ1–42among the different experimental groups (data not shown).

### The Effects of Interaction Between Different Dietary n-6/n-3 PUFA Ratios and n-3 LC-PUFA Content With Gonadal Status on the Expression of GFAP, Signaling Proteins and Synaptic Markers in the Cerebral Cortex of Female APP/PS1 Mouse

**FIGURE 5:**
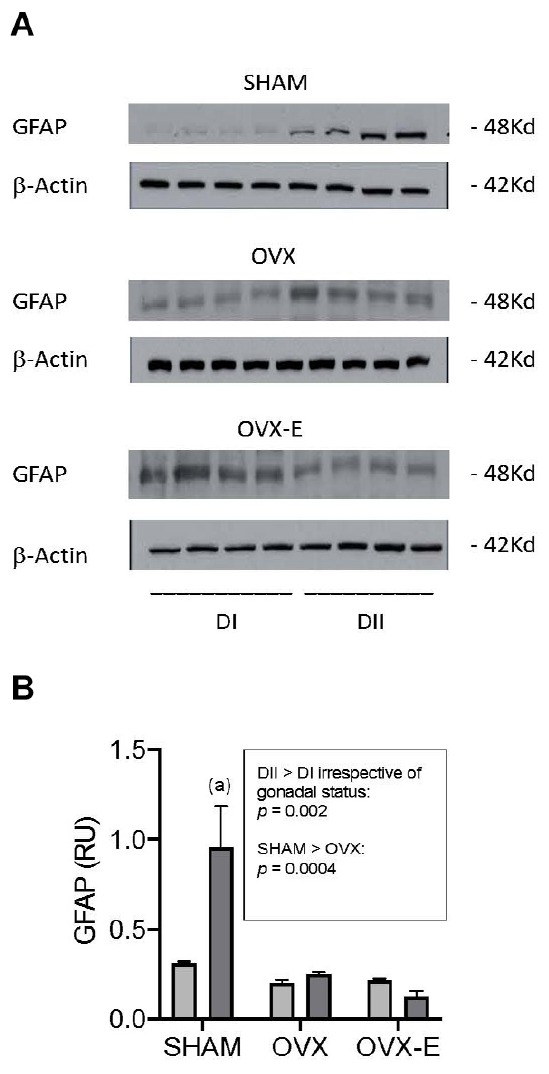
Effect of different dietary composition on GFAP expression in the cerebral cortex of female APP/PS1 mice under different gonadal conditions. **(A)** Western blots of GFAP and β-Actin. **(B)** Vertical axis represents relative units of densitometric quantification as mean ± SEM of four mice per group, normalized with respect to the loading control, β-Actin. Clear bars correspond to high n-6/n-3 ratio (DI), and dark bars correspond to low n-6/n-3 ratio (DII). Horizontal axis indicates the different gonadal status as follows: SHAM, intact sham-operated; OVX, ovariectomized, placebo-treated; OVX-E, ovariectomized, estradiol-treated. General ANOVA: *p* = 0.0004; (a) *p* = 0.0001.

The expression of GFAP and of selected signaling proteins and synaptic markers was compared between the cerebral cortex of animals fed DI and DII (animals maintained on SF were not included in these studies). When the dietary-induced changes in the astrocyte marker GFAP were quantified in Western blots (Figure 5A), more GFAP was detected in sham operated female APP/PS1 mice fed DII than in those fed DI (*p* = 0.002). This marked difference was not observed in ovariectomized animals, irrespective of whether they receive the placebo or estradiol. In addition, GFAP expression in all the intact animals (SHAM) was found to be higher than in the ovariectomized mice, irrespective of the diet (*p* = 0.0004) (Figure 5B).

**FIGURE 6:**
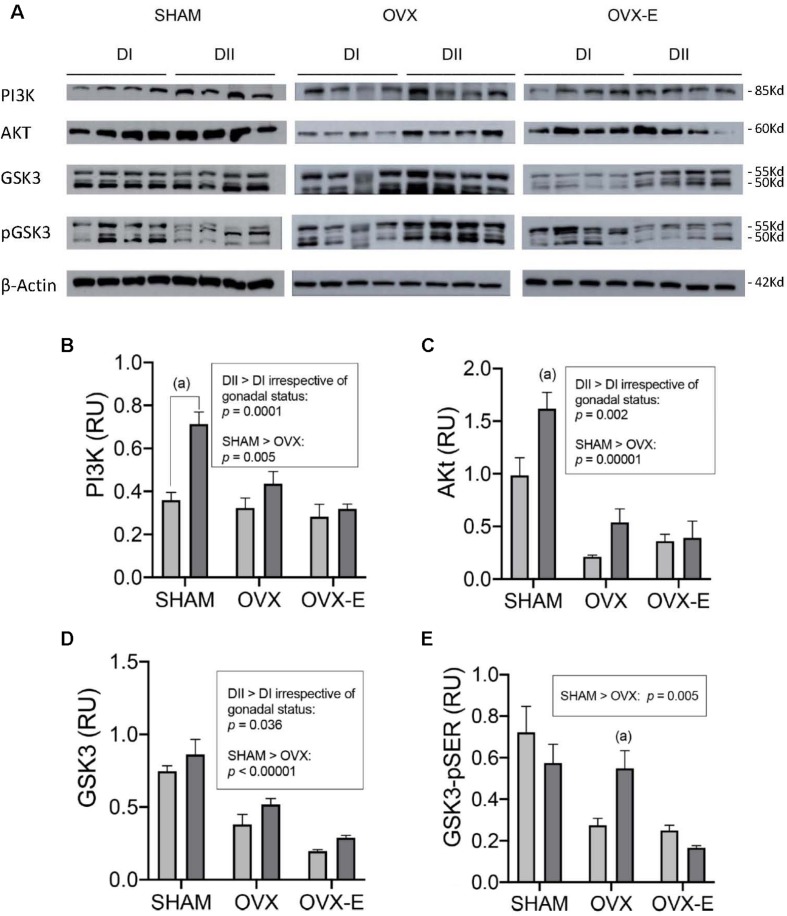
Effect of different dietary composition on the expression of signaling proteins in the cerebral cortex of female APP/PS1 mice under different gonadal conditions. **(A)** Western blots for PI3K, AKT, GSK3, GSK3-pSer (p-GSK3), and β-Actin. **(B–E)** Vertical axis represents relative units of densitometric quantification as mean ± SEM of four mice per group, normalized with respect to the loading control, β-Actin. Clear bars correspond to high n-6/n-3 ratio (DI), and dark bars correspond to low n-6/n-3 ratio (DII). Horizontal axis indicates the different gonadal status as follows: SHAM, intact sham-operated; OVX, ovariectomized, placebo-treated; OVX-E, ovariectomized, estradiol-treated. **(B)** General ANOVA: *p* = 0.000001; (a) *p* = 0.00001; Global effect of DII vs. DI: *p* = 0.0003. **(C)** General ANOVA: *p* = 0.02; (a) *p* = 0.0004; Global effect of DII vs. DI: *p* = 0.01. **(D)** General ANOVA: *p* = 0.02; (a) *p* = 0.03; (b) *p* = 0.008; Global effect of DII vs. DI: *p* = 0.002. **(E)** General ANOVA: *p* = 0.0004; (a) *p* = 0.0005.

In terms of signaling proteins (Figure 6A), such as the p85 subunit of PI3K, Akt, and GSK3 (total and serine phosphorylated), their quantitative expression in Western blots was normalized to that of Actin (Figures 6B–E). Sham-operated animals fed DII expressed PI3K (*p* = 0.0001) and Akt (*p* = 0.003) more strongly, yet these differences in the effect of diet were not observed in ovariectomized mice treated with either placebo or estradiol. However, the statistical analysis revealed a global enhancement in PI3K (*p* = 0.0001), Akt (*p* = 0.002), and GSK3 (*p* = 0.036) in the mice that received DII relative to those that received DI, irrespective of their gonadal status. In addition, PI3K (*p* = 0.005), Akt (*p* = 0.00001), GSK3 (*p* < 0.00001), and GSK3-pSer (*p* = 0.005) were expressed more strongly in intact sham-operated than in ovariectomized mice. This global effect of ovariectomy was not prevented or reverted by chronic estradiol administration since there were no significant differences when OVX mice were compared with OVX-E mice. As an exception to this general picture, DII significantly increased the GSK3-pSer levels in OVX females relative to those that received DI (*p* = 0.0005), an effect that was prevented by chronic estradiol treatment.

**FIGURE 7:**
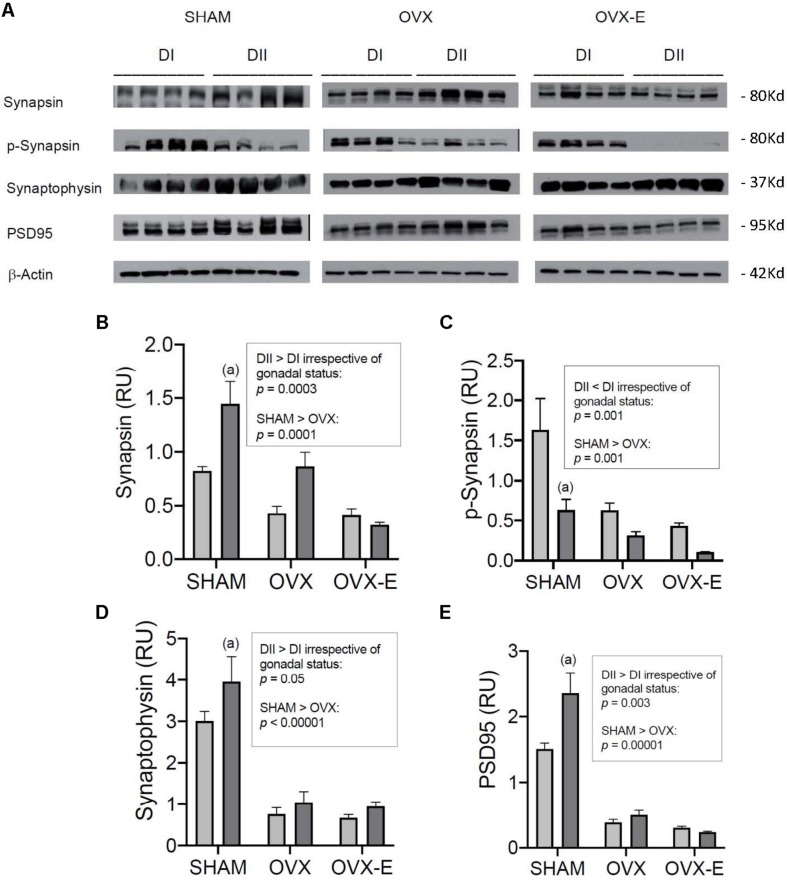
Effect of different dietary composition on the expression of synaptic proteins in the cerebral cortex of female APP/PS1 mice under different reproductive conditions. **(A)** Western blots for synapsin, p-synapsin, synaptophysin, PSD95, and β-Actin. **(B–E)** Vertical axis represents relative units of densitometric quantification as mean ± SEM of four mice per group, normalized with respect to the loading control, β-Actin. Clear bars correspond to high n-6/n-3 ratio (DI), and dark bars correspond to low n-6/n-3 ratio (DII). Horizontal axis indicates the different gonadal status as follows: SHAM, intact sham-operated; OVX, ovariectomized, placebo-treated; OVX-E, ovariectomized, estradiol-treated. **(B)** General ANOVA: *p* = 0.0005; (a) *p* = 0.01; (b) *p* = 0.001. **(C)** General ANOVA: *p* = 0.0006; (a) *p* = 0.003; (b) *p* = 0.01; (c) *p* = 0.0005; Global effect of DII vs. DI: *p* < 0.0000001. **(D)** General ANOVA: *p* = 0.0005; (a) *p* = 0.0006; (b) *p* = 0.003; (c) *p* = 0.009; Global effect of DII vs. DI: *p* < 0.0000001. **(E)** General ANOVA: *p* = 0.008; (a) *p* = 0.007.

We also analyzed the levels of some synaptic markers in the cerebral cortex, such as synapsin, p- synapsin, and synaptophysin, proteins involved in presynaptic mechanisms of neurotransmission, and PSD95 as a postsynaptic protein target (Figure 7A). Sham operated mice fed DII presented significantly higher levels of synapsin (*p* = 0.0008), synaptophysin (*p* = 0.035), and PSD95 (*p* = 0.0004) and lower levels of p-synapsin (*p* = 0.0007) compared to mice fed DI (Figures 7B–E). In the case of synapsin and p-synapsin, similar trends were observed in OVX mice although this trend did not reach significance. Nevertheless, a global effect of DII and DI was observed for synapsin (*p* = 0.0003), p-synapsin (*p* = 0.001), synaptophysin (*p* = 0.05), and PSD95 (*p* = 0.003) irrespective of gonadal status. Moreover, as for the signaling proteins, ovariectomy was associated with reduced synapsin (*p* = 0.0001), p-synapsin (*p* = 0.001), synaptophysin (*p* < 0.00001), and PSD95 (*p* < 0.00001) expression, independent of the diet. As mentioned above, this effect was not affected by chronic estradiol treatment.

## Discussion

Previous experimental (Calon et al., 2004; Lim et al., 2005; Green et al., 2007; Cao et al., 2009; Hooijmans et al., 2009) and epidemiological data (Kalmijn et al., 1997; Barberger-Gateau et al., 2002; Engelhart et al., 2002; Morris et al., 2005; Cederholm and Palmblad, 2010; Shinto et al., 2014) indicate that LC-PUFAs like DHA exert neuroprotective effects, preventing neurodegeneration and cognitive impairment. Indeed, diets enriched in DHA have been shown to reduce amyloidosis in AD mouse models (Lim et al., 2005; Green et al., 2007; Perez et al., 2010). In agreement with these studies, the results presented here indicate that a diet containing high levels of DHA and a low n-6/n-3 PUFA ratio reduces the amyloid Aβ1–40 in the hippocampus of female APP/PS1 mice when compared to diets with no traces of DHA and higher n-6/n-3 PUFA ratios. This effect we believe to be mainly due to increased 18:2n-6 supplementation. The effect of the DHA enriched diet on amyloid Aβ1–40 levels was accompanied by modified expression of components of a neuroprotective signaling pathway implicated in the AD pathology, the PI3K/Akt/GSK3 cascade and of key synaptic proteins like synapsin and PSD95 (Gomez-Sintes et al., 2011; Reddy, 2013; Llorens-Martin et al., 2014; Beurel et al., 2015). Thus, a DHA enriched diet increased the expression of synapsin and PSD95, the synapsin/p-synapsin ratio, and that of PI3K in sham APP/PS1 mice.

It is important to note that the levels of Aβ1–42, which is more prone to aggregate and form Aβ plaques, were not significantly modified by the diets. Thus, in the absence of an analysis of amyloid deposits it is not possible to determine the true implication of the diets in terms of Aβ plaque formation. However, PI3K is a neuroprotective kinase that promotes neuronal survival, memory, and cognition (Kitagishi et al., 2014; Arevalo et al., 2015; Matsuda et al., 2018). Similarly, PSD95 is an integral component of the postsynaptic density, which is involved in the development of new dendritic spines and that fulfills important roles in synaptic plasticity (Kim and Sheng, 2004; Ehrlich et al., 2007; Berry and Nedivi, 2017). Synapsins are a family of presynaptic proteins that play critical roles in synaptic development, neurotransmitter release, and neural plasticity through phosphorylation-dependent processes (Chi et al., 2003; Menegon et al., 2006; Giachello et al., 2010). Considering the roles of PI3K, PSD95, and synapsin, our results suggest that diets enriched in n-3 LC-PUFA and with different ratios of specific n-6 and n-3 PUFAs modulate the expression of key elements involved in neuroprotective signaling and synaptic transmission in AD mice, consistent with previous reports on the effects of DHA-enriched diets on hippocampal neuronal development (Cao et al., 2009), synaptic function (Cao et al., 2009), synaptic membrane proteins (Calon et al., 2004; Cansev et al., 2008; Sidhu et al., 2016; Herrera et al., 2018), and the restoration of neural plasticity and cognition after brain trauma (Wu et al., 2011). Our present findings indicate that two diets differing in their specific ARA, EPA, DPA, and DHA content, and in the n-6/n-3 PUFA ratio induce divergent changes in the lipidome of AD mouse brains. Compared to SF, DI increases the brain ARA and DPA levels while significantly reducing DHA levels, while DII caused a decrease in DPA and a significant increase in the DHA/DPA ratio. The different changes in ARA and DHA induced by the two diets are consistent with the differences in their composition because in rodents these lipids can be obtained either from the diet or they can be synthesized in the liver from linoleic acid (18:2n-6) or α-linolenic acid (18:3n-3), respectively (Sprecher, 2000; Williard et al., 2001; Gregory et al., 2011; Domenichiello et al., 2014). In humans, although DHA brain metabolism remains to be completely understood, the situation seems to be similar in that dietary intake of its precursors may be sufficient to supply the adult brain (Barceló-Coblijn and Murphy, 2009; Domenichiello et al., 2015).

The dietary effects on the lipid composition of the female APP/PS1 mouse brain detected here are in some aspects similar to those observed previously in WT mice maintained on the same diets (Herrera et al., 2018). To compare the results directly with those of our previous study on WT animals we used the same experimental diets (DI, DII) and SF (Herrera et al., 2018). The diets have a similar effect on the brain lipidome in both genotypes in terms of total FA, the relative levels of ARA, DPA, DHA, and the ratio of DHA/C22:5n-6. However, certain quantitative differences were observed. For instance, while the full repertoire of Ceramides, dh-Ceramides, Sphingomyelins, and dh-Sphingomyelins appeared to be elevated by both DI and DII diets in APP/PS1 mice, the effect of DII was significantly stronger than that of DI, in contrast to the effect observed in WT animals (Herrera et al., 2018). Equivalent lipidome remodeling has been reported in the hippocampus of female animals of the same AD model fed similar diets (Diaz et al., 2016), with WT and AD mice also suffering similar changes in the hippocampal lipid profile and certain differences depending on the genotype. Here, the main quantitative differences in the cerebral cortex between APP/PS1 mice and WT mice were found in complex lipids, suggesting that dietary composition may significantly affect lipid metabolism in the AD mouse brain.

Neuroprotective effects of ovarian hormones, in particular estradiol, are well characterized, and they include the downregulation of amyloidosis (Pike et al., 2009), the modulation of PI3K signaling (Marin et al., 2005; Arevalo et al., 2015), and the regulation of synaptic proteins like PSD95 (Luine et al., 2018) or synapsin (Rebas et al., 2005). All these parameters are modified by a DHA-enriched diet, as suggested in the present study, although the modification induced by the diet on synaptic proteins were not very large. Considering the hormonal decline associated with the menopause, an important unexplored question is whether ovarian function affects the neuroprotective effects of DHA, as suggested previously when a DHA enriched diet was seen to diminish Aβ plaque deposition in female but not male AD mice (Perez et al., 2010). Moreover, ovariectomy prevents the effects of this type of diet on the brain lipidome and on the protein profile of WT female mice (Herrera et al., 2018). Therefore, the main aim of the present study was to investigate the consequences of such an interaction between ovarian function and DHA-enriched diets in the AD mouse brain. Indeed, an interesting conclusion drawn from our results is that the dietary effects on the cerebral cortex lipidome in APP/PS1 mice differ depending on the levels of circulating estradiol. Thus, in ovariectomized AD animals fed DI, estradiol increased the brain levels of total FAs, DPA, Ceramides, dh-Ceramides, Sphingomyelins, and dh-Sphingomyelins. In addition, estradiol increased the DHA/DPA ratio in the brain of ovariectomized AD mice maintained on DII. These results extend those of our previous studies showing that ovariectomy profoundly alters the dietary effects on the lipidome in the cerebral cortex of WT mice (Herrera et al., 2018) and in the hippocampus of APP/PS1 mice (Diaz et al., 2016). However, one limitation of our study is that the lipidome of intact non-ovariectomized APP/PS1 animals was not analyzed. Nevertheless, these findings together indicate that the effect of diet on the brain lipidome is influenced by ovarian function and circulating estradiol levels in WT and AD mice. The influence of ovarian hormones on the effect of the diet on the mouse brain lipidome is consistent with human studies showing that estrogen stimulates the conversion of n-3 and n-6 precursors into their long chain metabolites (Decsi and Kennedy, 2011). Therefore, the potential synergies between diet and gonadal function should be carefully considered when designing dietary therapeutic approaches for neurological diseases.

As observed for the changes in the brain lipidome, the dietary effect on amyloid Aβ1–40 levels in APP/PS1 animals was also dependent on circulating estradiol, indicating that there is an interaction between the protective effect of estradiol and the intake of a DHA-enriched diet with a low n-6/n-3 PUFA ratio in an AD animal model. The effect of the DHA-enriched diet on the expression of some components of the PI3K/Akt/GSK3 pathway was also dependent on the hormonal status of AD mice. Thus, the effect of the DHA-enriched diet on the expression of Akt was lost in ovariectomized AD animals, while this diet only increased the GSK3 levels in ovariectomized animals. Furthermore, estradiol prevented the effect of the DHA-enriched diet on Akt and serine phosphorylated GSK3 in ovariectomized mice. Therefore, our present findings suggest that this hormone interacts with the DHA-enriched and a low n-6/n-3 PUFA ratio diet to regulate key components of this signaling pathway in the brain of AD mice. Similar conclusions can be drawn regarding the interaction of diet and estradiol on the regulation of synaptic proteins. Thus, the effect of the DHA-enriched diet on PSD95 and synapsin levels in the cerebral cortex of AD mice was prevented by ovariectomy or by estradiol administration, respectively.

The interaction of these two factors, diet and gonadal status, was also observed when analyzing the cerebral cortex levels of GFAP as an index of astroglial activation. Together, these results suggest that a diet enriched in DHA and a low n-6/n-3 ratio did not safeguard the beneficial effects on amyloid peptide accumulation, neuroprotective signaling, and synaptic function *per se*, independently of circulating estradiol. Only in combination with estradiol does a diet enriched in DHA and with a low n-6/n-3 ratio profoundly change Aβ metabolism in this AD model. However, in terms of the implications of our findings for AD, one limitation of the present study is that the APP/PS1 mouse model does not reproduce all the pathological hallmarks of the disease, such as tau pathology. Hence, further studies in other mouse models of AD will be necessary. Nevertheless, our study does support the idea that the design of dietary and/or pharmacological interventions for human neurodegenerative diseases should take into account the hormonal and metabolic status that may affect the therapeutic response.

## Data Availability Statement

The datasets generated for this study are available on request to the corresponding author.

## Ethics Statement

The animal study was reviewed and approved by the University of La Laguna Animal Care and Use Committee.

## Author Contributions

JH performed the animal experiments and was in charge of most experimental manipulations, hormone treatments, and collection of brain samples. JH and LO-G prepared the brain samples and performed the western blotting analysis. AM and GH participated in the experimental designs, animal treatments, and collection of samples. GF and JC performed the lipidomic analysis of brain samples. NA and CR analyzed the lipid composition of the experimental diets. CR collaborated in the interpretation of results of brain lipidome. LP-V performed the statistical analysis of quantitative results. RA conceived and designed the experiments and contributed together with LP-V to the design and interpretation of specific statistical analysis. RA, LG-S, and FW were in charge of the final interpretation of results and wrote the manuscript.

## Conflict of Interest

The authors declare that the research was conducted in the absence of any commercial or financial relationships that could be construed as a potential conflict of interest.
