# Supplementary material for: Ovarian Hormone-Dependent Effects of Dietary Lipids on APP/PS1 Mouse Brain
Source: Front Aging Neurosci. 2019 Dec 19;11:346. doi: 10.3389/fnagi.2019.00346 (PMC6930904; doi:10.3389/fnagi.2019.00346)
Supplement: Supplementary file 1 [file Table_1.pdf]

SUPPLEMENTARY TABLE 1

Densitometric data from Western blots of the different proteins analyzed. All values are normalized with respect to the loading control, β-Actin										
Experimental conditions		GFAP	PI3K	Akt	GSK3	GSK3-pSer	Synapsin	p-Synapsin	Synaptophysin	PSD95
SHAM	DI	0.2749	0.3392	0.6290	0.6559	0.4044	0.7059	0.5684	3.1042	1.2441
		0.3207	0.3381	0.7991	0.7700	1.0129	0.8521	1.7455	2.3620	1.5932
		0.3321	0.2964	1.1512	0.8376	0.7016	0.8698	2.4450	3.4808	1.6544
		0.3011	0.4642	1.3635	0.7100	0.7701	0.8703	1.7718	3.0756	1.5401
	DII	0.3604	0.5685	1.5375	0.5611	0.3417	0.9827	0.6319	2.8145	1.6854
		0.9494	0.7329	1.5432	0.9590	0.6788	1.2966	1.0062	5.1598	2.1537
		1.0302	0.7034	2.0584	0.8850	0.5312	1.5268	0.3966	3.0217	2.4011
		1.4837	0.8459	1.3290	1.0400	0.7470	1.9749	0.5013	4.8369	3.1830
OVX	DI	0.1710	0.3580	0.2005	0.3343	0.3343	0.3288	0.8040	0.5499	0.3130
		0.1830	0.2777	0.1751	0.3152	0.2152	0.3524	0.5840	0.7183	0.3520
		0.1740	0.2206	0.2507	0.2833	0.2186	0.4082	0.7470	0.5786	0.3630
		0.2580	0.4349	0.2223	0.5826	0.3299	0.6246	0.3910	1.2126	0.5280
	DII	0.2730	0.5660	0.5364	0.6315	0.3928	0.6078	0.2280	1.2640	0.4090
		0.2100	0.3025	0.2810	0.4465	0.4719	0.9288	0.4490	0.6118	0.5020
		0.2520	0.3848	0.4446	0.4731	0.7846	1.2036	0.3100	0.6361	0.7010
		0.2520	0.4881	0.8916	0.5230	0.5469	0.7262	0.2590	1.6712	0.3990
OVX-E	DI	0.2160	0.1696	0.2250	0.2273	0.2131	0.3087	0.4740	0.8131	0.2820
		0,2260	0.2211	0.5316	0.1910	0.3214	0.5858	0.5110	0.7832	0.3730
		0.1920	0.2991	0.3086	0.1912	0.2431	0.3535	0.3720	0.5330	0.2640
		0.2260	0.4379	0.3770	0.1711	0.2216	0.3932	0.3760	0.5696	0.3020
	DII	0.1690	0.3823	0.8368	0.2641	0.1554	0.3613	0.1080	0.8612	0.2740
		0.1420	0.3182	0.3269	0.2607	0.1448	0.2517	0.0950	0.8243	0.1990
		0.1720	0.2899	0,3043	0.3269	0.1793	0.3240	0.1100	0.9822	0.2420
		0.1530	0.2847	0.0987	0.3043	0.1859	0.3459	0.1090	1.2263	0.2380

SHAM: Intact, sham-operated controls
OVX: Ovariectomized, placebo-treated
OVX-E: Ovariectomized, estradiol-treated
